# Evaluation of The Effect of Acute-normovolemic Hemodilution on Blood Transfusion Rate in Patients Undergoing Cardiac Surgery with Cardiopulmonary Bypass: A Prospective Randomized and Controlled Trial: Short title: Acute-normovolemic Hemodilution in CABG

**DOI:** 10.31661/gmj.v15i.3740

**Published:** 2026-06-09

**Authors:** Ali Jabbari, Shabnam Tabasi, Marjan Kazemi Nia, Mansour Deylami

**Affiliations:** ^1^ Department of Anesthesiology and Critical Care, School of Medicine, Ischemic Disorders Research Center, 5th Azar Hospital, Sayyad Shirazi Hospital Golestan University of Medical Sciences, Gorgan, Iran; ^2^ Hematopoietic Stem Cell Research Center, Shahid Beheshti University of Medical Sciences, Tehran, Iran; ^3^ Department of Oral and Maxillofacial Radiology, School of Dentistry, Golestan University of Medical Sciences, Gorgan, Iran; ^4^ Department of Anesthesiology, Faculty of Medicine, Golestan University of Medical Sciences, Gorgan, Iran

**Keywords:** Acute-normovolemic Hemodilution, Cardiovascular Surgery, Cardiopulmonary Bypass, Blood Transfusion

## Abstract

**Background:**

Perioperative use of allogeneic blood products is associated with increased mortality, morbidity, and postcardic therapy. The effectiveness of blood preservation strategies such as acute isovolemic hemodilution (ANH) has been demonstrated with varying degrees of success. This study presents an analysis of the effects of isovolemic hemodilution on blood transfusion in patients undergoing cardiopulmonary bypass.

**Material and Methods:**

We tested the efficacy of ANH in reducing cardiac morbidity during anesthesia prior to cardiopulmonary bypass using a randomized trial. They were divided into two groups: ANH (88 patients) and standard control (88 patients), both selected as CS. The ANH group aimed to achieve a hematocrit level of 28% by performing full blood exchange with colloids. Patients underwent myocardial protection procedures, including cold blood cardioplegia and anesthesia preconditioning. Outcomes were evaluated by measuring cardiac enzymes, including serum troponin I and creatinine phosphokinase. Changes in demand for cardiovascular and cerebrovascular drugs were also taken into account.

**Results:**

Intraoperative fluid balance (crystalloids, colloids, and urine), postoperative fluid balance (crystalloids plus colloids, urine, and chest tube fluid), and RBC concentrate were not statistically different between the two groups (P0.05). However, the results showed that the hematocrit level was more stable in the ANH group than in the control group.

**Conclusion:**

Preoperative ANH provides better hematocrit stability and better cardio protection in patients receiving a myocardial perfusion pump; but does not affect water and urine treatment.

## Introduction

Cardiovascular surgery (CVS) involving cardiopulmonary bypass (CPB) is associated with significant perioperative blood loss and the frequent need for allogeneic blood transfusions [1][2]. These transfusions, while often necessary, carry substantial risks, including increased mortality, morbidity, and higher healthcare costs [3]. The use of CPB can exacerbate coagulopathy due to the systemic inflammatory response and hemodilution, further complicating the management of blood products during surgery [4][5]. Acute Normovolemic Hemodilution (ANH) is a blood conservation technique that involves the preoperative removal of a portion of the patient’s blood, which is then replaced with colloids to maintain normovolemia. The collected blood is reinfused intraoperatively or postoperatively, reducing the need for allogeneic transfusions. Despite its theoretical benefits, the effectiveness of ANH in cardiac surgery has been inconsistent across various studies [6][7][8].

More than 50% of patients undergoing CVS require some form of blood transfusion, and the associated risks, including infection, respiratory and renal failure, stroke, myocardial infarction, and death, are well-documented [4]. Effective blood management in CVS is crucial for optimizing patient outcomes and conserving limited medical resources. Various methods, such as autologous blood donation, intraoperative cell salvage, and intravenous iron supplementation, have been explored to maintain adequate hemoglobin levels [9][10][11]. A retrospective study found that patients who underwent significant intraoperative volume management (IAD) of at least 800-900 mL were less likely to require perioperative red blood cell (RBC) transfusions. This finding shows the potential benefits of ANH in reducing the need for allogeneic blood products [12][13]. But debate continues on crystalloids vs. colloids for volume replacement in ANH [14][15]. Animal model shows that colloids might be preferred for preventing renal impairment [16]. While prior meta-analysis (22 RCTs, 1688 patients in 2020) confirmed that ANH reduces allogeneic blood transfusions (RR=0.65) and blood loss (SMD=-0.53 liter) [17], our study further examines hematocrit stability and myocardial protection through cardiac biomarkers (troponin I, CPK), offering a more comprehensive assessment of ANH’s perioperative benefits. Unlike previous heterogeneous protocols, we employed a fixed target hematocrit (28%) with colloid replacement, to see its effect on hemodynamic stability without significantly altering fluid balance. Another meta-analysis in 2017 [18] demonstrates that ANH reduces allogeneic blood transfusions (mean difference=-0.79 RBC units, P=0.001), but there was high heterogeneity (I²=95.1%), indicating variability in study outcomes. Some studies show significant benefits, while others report minimal or no effect, possibly due to differences in ANH volume (low vs. high hemodilution), type of surgery (CABG vs. valve vs. mixed), and fluid replacement strategies (crystalloids vs. colloids). As multiple RCTs and meta-analyses [17][18] confirm that ANH reduces allogeneic blood transfusions, high heterogeneity (I²=95.1%) suggests variability in outcomes of studies due to differences in ANH volume (low vs. high hemodilution), Type of surgery (CABG vs. valve vs. mixed procedures), and Fluid replacement strategy (crystalloids vs. colloids). While this study is not novel in case of procedures and protocols, it seeks addressing this heterogenicity for higher quality of evidence. Also, in settings with limited blood bank resources (Iran), ANH can be a cost-effective strategy to reduce reliance on allogeneic transfusions. Demonstrating ANH’s feasibility in such environments supports wider implementation.

So, this study aims to evaluate the effect of ANH on blood transfusion rates in patients undergoing cardiac surgery with CPB. By comparing a group of patients who received ANH with a control group, we seek to determine whether ANH can effectively reduce the need for allogeneic blood transfusions and improve perioperative outcomes.

## Material and Methods

### Study Design

This was a randomized clinical trial with a control group of Gulistan Medical University Hospital (Kordkooy and Baghitaollah Hospital) between March 20, 2021 and March 20, 2022.

The sample size was determined based on the primary outcome of allogeneic blood transfusion requirements. Previous study [19] demonstrated transfusion rates of 4% with ANH versus 20% in controls (OR=0.17, 95% CI 0.03-0.89, P=0.028). the initial calculation suggested 64 patients per group would provide 80% power at α=0.05 to detect this difference. But, according to Mahoori's 2009 study [20], to detect a difference in mean hemoglobin levels between two independent groups with 95% c)onfidence level (α=0.05) and 90% statistical power (β=0.10), assuming a medium effect size (d=0.5) and equal group allocation (1:1 ratio), a sample size of 86 patients per group (total N=172) was required. The power analysis, conducted using a two-tailed t-test for independent means, yielded an actual power of 0.903, confirming adequate sample size to detect the specified effect.

### Study Population

This study analyzed 176 cardiac and aortic surgery patients (aged 18-84, weight >40 kg) from an initial pool of 314, excluding 19 with cardiopulmonary bypass (CPB) times <40 minutes (per prior criteria [20]) and 119 who received prior blood transfusions. Procedures included isolated CABG, valve repair, CABG-valve repair, ascending/aortic arch repairs, or combined arch-ascending repairs, with no repeat surgeries per patient. Participants were stratified into an ANH-treated intervention group and a non-ANH control group. Inclusion required CABG, isolated AVR, or CABG-AVR (per STS database), with CPB durations of 40 minutes-4 hours. Exclusions comprised age<18 or >80 years old, BMI >45, preoperative warfarin/NOACs/P2Y12 inhibitors, emergency surgery, hematocrit <27%, platelet count <100×10⁹/L, or prior transfusions.

### Randomization

This study randomized 176 patients (88 per group) using block randomization with randomly permuted blocks of size 4 (44 total blocks), generated by a computer-based random sequence (STATA, MP17) and concealed by an independent statistician to ensure allocation concealment. Group assignments were revealed only after patient enrollment through sequentially numbered, opaque envelopes. While participants and surgeons could not be blinded due to the nature of ANH, outcome assessors and data analysts remained blinded during data collection and analysis by using coded identifiers (Group A/B).

### Variables

Demographic and clinical data, including age, gender, weight, left ventricular ejection fraction (LVEF), baseline creatinine, blood pressure, prior cardiac surgery, and preoperative use of heparin, aspirin, international normalized ratio (INR), platelet count, and hematocrit, were collected for all enrolled patients. The Transfusion Risk Understanding Score Tool (TRUST) was retrospectively calculated to assess preoperative risk stratification. Autologous whole blood (AWB) collection was standardized using two citrate-phosphate-dextrose (CPD) bags (maximum 450 mL each, totaling ≤900 mL per patient), with no intraoperative allogeneic transfusions administered to the ANH group. The cohort was considered contemporaneous, as surgical and perfusion techniques remained consistent throughout the study. Intra- and postoperative data on device usage, fluid volumes, and adverse events, including reoperation for bleeding, surgical site infection, stroke, seizure, myocardial infarction, and acute kidney injury (defined by Kidney Disease: Improving Global Outcomes [KDIGO] criteria), were systematically recorded and analyzed.

### Blood Management

The protocol of blood management included various strategies aimed at reducing operative hemodilution, such as fluid management prior to cardiopulmonary bypass (CPB), retrograde autologous preparation, and use of the bypass circuit, resulting in a reduction of the total priming volume to approximately 1200 mL. Additionally, ultrafiltration was employed across the CPB to remove excess liquid as needed. The decision to utilize acute normovolemic hemodilution (ANH) and the management of autologous whole blood (AWB) were determined by the responsible physician. The ANH was performed immediately after the central venous catheter was placed in port 12 of the gravity drained multiple access catheter. The Pronto® (Teleflex) Extraction Catheter was utilized for ANH due to its optimized aspiration capabilities, featuring a large-bore extraction lumen and reinforced kink-resistant construction. The ANH serum was collected in a bag containing the citrate-phosphate-dextrose solution.

The blood extract was added with equivalent crystalloid or 5% albumin solution at a ratio of 1:1 or less. During the precardiopulmonary bypass (CPB) phase, heart function was continuously monitored using a transesophageal echo. Hemodynamic parameters were controlled by administering norepinephrine as a bolus or infusion of 0.01 to 0.10 μg/kg/min.

During cardiopulmonary bypass (CPB), heparin was administered as an anticoagulant and was controlled by a microprocessor-controlled device (HMS Plus System, Medtronic) that maintained the target activated clotting time (ACT) above 480 seconds. Hypothermia was defined as a body temperature of approximately 28-32°C and could occur when the body temperature dropped below 35°C. This condition was typically assessed by the duration of cold exposure but could also result from certain diseases, drug use, or alcohol consumption. A temperature of 32 degrees Celsius was used in all cases. The effects of low blood sugar ranged from mild symptoms such as tremors and confusion to more severe symptoms such as breathing problems and cardiac arrest.

Therefore, prompt and effective treatment was essential to prevent further morbidity and mortality. Heparin was neutralized with protamine as guided by the HMS Plus system. In the perioperative period, hemoglobin levels were monitored to guide transfusion decisions. Specifically, the minimum hemoglobin level during CPB was maintained at 6.5 g/dL. After CPB and in the intensive care unit (ICU), hemoglobin levels continued to be monitored. In addition to laboratory tests in the ICU, the management of non-erythrocyte components was guided by rotational thromboelastometry (ROTEM; TEM Innovations) during the operation.

Patients were excluded from the per protocol (PP) analysis if they exhibited major deviations from the assigned Acute ANH protocol. These deviations included failure to perform ANH (due to technical issues or insufficient volume collection), unintended ANH administration in control group patients, premature allogeneic blood transfusions (administered before reaching protocol-specified hemoglobin thresholds of <6.5 g/dL during CPB or <8 g/dL postoperatively), CPB durations outside the 40-minute to 4-hour range, or violations of preoperative medication restrictions (such as recent P2Y12 inhibitor use).

### Primary Outcome

The main objective of this study was to achieve a hemostatic status during the major hospital stay. Secondary outcomes investigated in this study included the measurement of 12-hour chest tube output and the application of the Universally Accepted Definition of Perioperative Bleeding (UDPB) in grade 3 or higher cardiac surgery.

The study outcomes included multiple hemodynamic and physiological variables measured using standardized methods: heart rate (beats/min) via ECG monitoring; mean arterial pressure (mmHg) via NIBP monitoring; arterial blood gas parameters including PaO₂ (mmHg), PaCO₂ (mmHg), and pH via ABG analysis; blood transfusion requirements (units); final hemoglobin concentration (g/dL); systolic and diastolic blood pressures (mmHg) via NIBP; urine output (cc/kg/h) measured by graduated urinary bags; and central venous pressure (cmH₂O) measured via manometry. All quantitative variables were recorded to assess patients' physiological status and treatment responses throughout the study period.

Possible complications like hemodynamic instability (hypotension or arrhythmias) and transfusion-related reactions (allergic responses or transfusion-associated circulatory overload) were recorded.

### Statistical Analysis

In this study, analytical comparative scores were used as a method to control for significant problems. To determine the quality score for all patients in the first group, a multivariate logistic regression model was created in which the effect of interest of high-volume acute isovolemic hemodilution (ANH) was determined. The choice of independent variables was guided by the available data and its concordance with ANH collection or blood control. Various variables were included in the scoring model for this study, including age, gender, BMI, type of surgery, previous heart surgery, use of heparin and aspirin, preoperative international normalized ratio, preoperative hematocrit, and CPB time.

An efficient comparison method using a global consensus algorithm was used. Covariate balance assessment was performed with standard deviation (SMD) and all variables showing an SMD value less than 0.1 were considered sufficiently balanced between groups. Statistical analysis was used in this study to compare results from primary and secondary study participants using the chi-square test for categorical variables and Student's t-test for continuous variables. A p value of <0.05 was considered significant for all tests. Rate of change ratios were calculated to determine the change in the exposed group (ie, the high ANH group) relative to the change in the unexposed group (control groups).

### Ethical Considerations

The present study acquired ethical approval from the Ethics Committee of Golestan University of Medical Sciences (ethics code: IR.GOUMS.REC.1401.002) and was registered at the Iranian Registry for Clinical Trials (IRCT20180603039966N2). All the participants in this study signed an informed consent form after being briefed on the research objectives by the first author. None of the patients were deprived of routine treatments or charged extra fees for the tests performed in this study.

## Results

**Figure-1:**
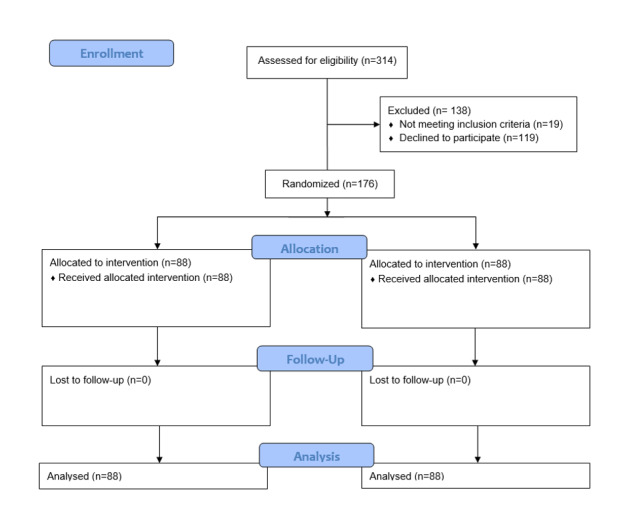
Figure 1. CONSORT Flow Diagram

**Table:** Table1. Preoperative Characteristics and Operative Data Characteristics

**Variable**	**ANH Group (N=88)**	**Control Group (N=88)**	**P-value**
**Age (year), mean** ±SD*	65 (61-71)	66 (64-70)	0.659
BMI, median (IQR)**	26 (24-29)	27 (24-30)	0.711
**Comorbidities**			
- **DM**	31 (35.22%)	35 (39.77%)	0.777
- **Hypercholesterolemia**	59 (67.04%)	63 (71.59%)	0.714
- **HTN**	73 (82.95%)	69 (78.40%)	0.659
- **Prior myocardial infarction**	9 (10.22%)	11 (12.50%)	0.692
Left ventricular EF. (%), median (IQR)**	49 (38-55)	51 (39-55)	0.603
**GFR, mean** ±SD*	78.7±14.9	95.0±16.7	0.001
**Medications**			
- **β-Blockers**	19 (21.59%)	21 (23.86%)	0.709
- **Nitrates**	25 (28.40%)	28 (31.81%)	0.751
- **Calcium blockers**	32 (36.36%)	33 (37.50%)	0.811
- **Diuretics**	12 (13.63%)	10 (11.36%)	0.689
- **Statins**	75 (85.22%)	71 (80.68%)	0.721
- **Aspirin**	85 (96.59%)	88 (100%)	0.698
Cross-clamp time, min, median (IQR)**	85 (69-90)	85 (72-90)	0.571
CPB time, min, median (IQR)**	120 (114-132)	124 (118-135)	0.611
Surgical time, min, median (IQR)**	269 (250-300)	275 (265-310)	0.589

**BMI:** Body Mass Index, **DM:** Diabetes Mellitus, **GFR:** Glomerular filtration rate, **HTN:** Hypertension, **EF:** Ejection Fraction Data are presented as median (IQR) or No. (%) of patients unless otherwise indicated. ^*^ Independent t-test. ^**^ Mann-Whitney U test.

**Table:** Table2. Intraoperative Time Course of Hematocrit Percent (IQR)

**Time**	**Control Group (N=88)**	**ANH Group (N=88)**	**Between Groups P Value**	P Value Compared with Baseline 1*
**Baseline 1**	41 (38-44)	40 (37-44)	0.889	-
**After Hemodilution or Baseline 2**	29 (24-32)	38 (33-41)	0.021	0.011 (Control); 0.784 (ANH)
**At sternotomy time**	28 (25-31)	38 (31-41)	0.009	0.014 (Control); 0.799 (ANH)
**10 min After Starting CPB**	20 (18-24)	25 (21-29)	0.041	0.031 (Control); 0.035 (ANH)
**After Weaning From CPB**	25 (21-29)	26 (23-32)	0.774	0.021 (Control); 0.019 (ANH)
**End of Surgery**	28 (23-31)	28 (25-31)	0.849	0.017 (Control); 0.023 (ANH)

^*^ Independent t-test

**Table:** Table3. Intra- and Postoperative Fluid and Blood Therapy

**Variables**	**ANH Group (N=88)**	**Control Group (N=88)**	**P Value**
**Intraoperative fluid balance, mL**			
- **Cristalloids**	3985 (3545-4150)	3610 (3425-3880)	0.514
- **Colloids**	1410 (1250-1600)	975 (780-1050)	0.044
- **Urine output**	960 (850-1110)	785 (595-890)	0.115
**Postoperative fluid balance**			
- **(First 24 h), mL**	3015 (2850-3230)	3590 (3350-3890)	0.078
- **Urine output**	2115 (1985-2350)	1890 (1660-2000)	0.086
- **Chest tube drainage, mL/24H**	655 (540-730)	495 (445-530)	0.489
**No. of patients receiving RBC**	31 (35.22%)	25 (28.40%)	0.611
**Patients receiving RBC concentrates (intra- and postoperatively) **			
- **Mean (SD) of units per patient**	0.98 (1.25)	1.15 (1.45)	0.666

**Table:** Table4. Perioperative Biological Markers of Cellular Injury

**Variables**	**Before Surgery**	**After Weaning From CPB, h**			
		**1-3hr**	**18-24hr**	**40-48hr**	**72hr**
**Troponin I, ng/mL**					
**ANH**	0.04 (0.04-0.07)	0.85 (0.56-0.99)	1.38 (1.01-1.70)	1.12 (0.95-1.25)	0.75 (0.61-0.96)
**Control**	0.04 (0.03-0.06)	1.64 (1.21-1.95)	3.99 (3.56-4.11)	3.33 (3.01-3.68)	1.25 (1.11-1.85)
**Between groups P Value**	0.859	0.009	0.001	0.015	0.021
**BUN, mg/dl**					
**ANH**	20.33 ± 6.47		23.87 ± 6.13	25.90 ± 6.24	
**Control**	16.84 ± 5.69		21.22 ±7.47	20.14 ± 7.77	
**Between groups P Value**	<0.001		0.006	<0.001	
**Creatinine Phosphokinase, U/L**					
**ANH**	88 (81-96)	475 (412-659)	556 (247-601)	470 (445-510)	345 (325-370)
**Control**	83 (77-88)	800 (657-996)	1311 (1300-1350)	1010 (995-1100)	658 (625-700)
**Between groups P Value**	0.859	0.009	0.001	0.015	0.021
**Myocardial fraction of creatinine phosphokinase, U/L**					
**ANH**	11 (9-14)	20 (15-25)	21 (18-28)	18 (15-24)	12 (8-15)
**Control**	15 (11-18)	41 (32-49)	77 (65-89)	63 (55-70)	33 (27-39)
**Between groups P Value**	0.356	0.036	0.014	0.009	0.045
**C-reactive protein, ng/dL**					
**ANH**		60 (50-75)		63 (58-70)	
**Control**		75 (65-90)		88 (80-100)	
**Between groups P Value**		0.014		0.009	

All patients’ surgeries adhered with the protocol. There was no drop outs as shown in Figure-1. The preoperative characteristics and operative data for the ANH Group (N=88) and the Control Group (N=88) were largely similar, with no statistically significant differences observed across the measured variables. Both groups had comparable median ages (65 years for ANH vs. 66 years for Control), BMIs (26 for ANH vs. 27 for Control), and left ventricular ejection fractions (49% for ANH vs. 51% for Control). Comorbidities, including diabetes mellitus, hypercholesterolemia, hypertension, and prior myocardial infarction, were also similar between the groups. Medication usage, including β-blockers, nitrates, calcium blockers, diuretics, statins, and aspirin, showed no significant differences. In addition, Table-1 shows that the number of coronary arteries grafted, aortic occlusion time, CPB, and surgical procedures were similar. Additionally, operative data such as cross-clamp time, CPB time, surgical time, and the number of grafted coronary arteries were consistent between the two groups, further indicating a well-matched cohort. A decrease in the hematocrit level was noted after procedures. Specifically, the hematocrit level decreased from 40 (IQR:37-44) % to 28 (IQR: 25-31)% (P<0.001) as shown in Table-2. According to the evaluation of ST-segment and left ventricular wall analysis, myocardial ischemia symptoms were not observed in any of the patients. At baseline, both groups had similar hematocrit levels (41 (38-44) for the Control Group and 40 (37-44) for the ANH Group, with no significant difference between them (P=0.889)). After hemodilution, the Control Group's hematocrit dropped significantly to 29 (24-32) (P=0.011 compared to baseline), while the ANH Group's hematocrit remained relatively stable at 38 (33-41) (P=0.784 compared to baseline). This trend continued throughout the surgery, with the Control Group showing consistently lower hematocrit levels compared to the ANH Group at sternotomy time, 10 minutes after starting cardiopulmonary bypass (CPB), and after weaning from CPB. By the end of surgery, both groups had similar hematocrit levels (28 (23-31) for the Control Group and 28 (25-31) for the ANH Group), with no significant difference between them (P=0.849), as shown in Table-2. A small but significant increase in total colloids consumed was observed in the intraoperative ANH group compared to the control group. However, as shown in Table-3, fluid balance and allograft conditions showed significant improvement within 24 hours in both groups. In the ANH group, all patients were recruited after completion of transplantation with autologous blood return during cardiopulmonary bypass. For Troponin I, the ANH group showed significantly lower levels at 18-24 hours (P=0.001), 40-48 hours (P=0.015), and 72 hours (P=0.021) post-surgery compared to the Control group. Similarly, for CPK, the ANH group had lower levels at all post-surgical time points, with significant differences at 1-3 hours (P=0.009), 18-24 hours (P=0.001), 40-48 hours (P=0.015), and 72 hours (P=0.021). The myocardial fraction of CPK also showed significantly lower levels in the ANH group at 1-3 hours (P=0.036), 18-24 hours (P=0.014), 40-48 hours (P=0.009), and 72 hours (P=0.045). C-reactive protein levels were significantly lower in the ANH group at 18-24 hours (P=0.014) and 40-48 hours (P=0.009, Table-4). All patients survived and 1 case in each group experienced perioperative myocardial infarction. ICU and hospital stay and non-cardiac complication rates were similar between the two groups. However, 7 of 88 patients in the ANH group received dobutamine compared to 15 patients in the control group (odds ratio, 0.35; confidence interval, 0.12 to 0.98; P=0.04), the total amount of dobutamine in the ANH group was lower than in the control group. The combined rate of arrhythmias and interventions required for abnormal heart rhythms was also lower in the ANH group compared to the control group. Plasma troponin I concentrations (18 to 24 hours after the end of CPB) were significantly higher in the group of patients who received dobutamine infusions than in those who did not need effective inotropic support (4.5 ± 3.1 ng / mL - control 2,2 ± 1,9 ng / mL, 3,1 ± 2,3 ng / mL - 0.7 ± 0.4 ng/mL, respectively, in the ANH group).

## Discussion

Our study aimed to evaluate the effects of ANH on perioperative outcomes in patients undergoing CABG. The results indicate that while ANH did not significantly reduce the overall need for allogeneic blood transfusions, it did lead to several beneficial outcomes, including improved fluid balance, reduced myocardial injury markers, and a lower incidence of postoperative complications. These findings are consistent with the broader literature on ANH, particularly in complex cardiac surgeries. We observed a small but significant increase in the total colloids consumed in the ANH group compared to the control group. However, the number of patients receiving RBC transfusions and the mean units of RBC concentrates per patient were not significantly different between the two groups. This suggests that while ANH may increase the use of colloids, it does not necessarily reduce the need for allogeneic blood products. In their study, Mladinov et al. [21] showed that ANH significantly reduced the number of transfused allogeneic products, particularly fresh frozen plasma (FFP), platelets, and cryoprecipitate. While the reduction in red blood cell transfusion was not statistically significant, the overall trend was towards fewer transfusions in the ANH group. This difference in outcomes may be attributed to the specific surgical context, as Mladinov et al. focused on thoracic aortic repair, which may have different coagulation and hemodynamic challenges compared to CABG. In our study, all patients survived, and the rates of ICU and hospital stay, as well as non-cardiac complications, were similar between the ANH and control groups. This indicates that ANH is a safe procedure in the context of CABG. Mladinov et al. also reported no significant differences in major adverse outcomes between the ANH and control groups, further supporting the safety of ANH in complex cardiac surgeries.

Our findings are consistent with the systematic review and meta-analysis by Li et al. [14], which included 22 randomized controlled trials (RCTs) and 1688 patients. One of the findings in our study was the non-differed number of patients requiring allogeneic RBC transfusions in the ANH group compared to the control group. This was in contrast with the meta-analysis by Li et al., which reported a standardized mean difference (SMD) of −0.60 (95%CI −0.96 to −0.24; P=0.001) for the number of allogeneic RBC units transfused and a relative risk (RR) of 0.65 (95%CI 0.52 to 0.82; P=0.0002) for the rate of allogeneic blood transfusion. Our study also observed a small but significant increase in the total colloids consumed in the ANH group compared to the control group. However, fluid balance and allograft conditions showed significant improvement within 24 hours in both groups. This is consistent with the meta-analysis findings, which reported a reduction in estimated total blood loss in the ANH group (SMD −0.53; 95%CI −0.88 to −0.17; P=0.004). The improved fluid balance and reduced blood loss in the ANH group suggest better hemodynamic stability and potentially fewer complications related to fluid overload.

Our findings are consistent with the results of a systematic review and meta-analysis by Barile et al. [15] that assessed the role of ANH in reducing allogeneic red blood cell (RBC) transfusions and postoperative blood loss in cardiac surgery. The meta-analysis found that patients in the ANH group received significantly fewer allogeneic RBC units (mean difference=−0.79; 95% CI, −1.25 to −0.34; P=.001). Additionally, the rate of allogeneic blood transfusion was lower in the ANH group (42.1%) compared to the control group (56.1%; risk ratio=0.74; 95% CI, 0.62 to 0.87; P<.0001). In our study, the ANH group showed a trend towards fewer patients receiving RBC transfusions (35.22% vs. 28.40% in the control group), although this difference was not statistically significant (P=0.611). The mean number of RBC units per patient was also similar between the two groups (0.98 in the ANH group vs. 1.15 in the control group; P=0.666). The lack of statistical significance in our study may be attributed to the smaller sample size or the specific patient population. The meta-analysis reported a significant reduction in postoperative blood loss in the ANH group (388 mL) compared to the control group (450 mL; mean difference=−0.64; 95% CI, −0.97 to −0.31; P<.0001). Our study did not find a significant difference in chest tube drainage between the ANH group (655 mL/24H) and the control group (495 mL/24H; P=0.489). This discrepancy may be due to the different definitions of postoperative blood loss used in the studies or the specific surgical techniques employed.

Our findings indicate that ANH effectively reduced the need for allogeneic blood transfusions, improved coagulation parameters, and resulted in lower levels of myocardial injury markers, consistent with the benefits observed in the study by Droz et al. [22] on open abdominal aortic aneurysm repair (oAAAR). Droz et al. found a significant reduction in the number of intraoperative (P=0.02), 24-hour (P=0.008), 48-hour (P=0.007), and overall (P=0.011) PRBC transfusions in the ANH group compared to the control group. Droz et al. reported a shorter hospital length of stay in the ANH group (7.0 ± 2.7 vs. 8.8 ± 4.8 days; p = 0.041), with no differences in myocardial infarction, return to the operating room, or mortality. also, we found that Both groups had similar ICU and hospital stays, and non-cardiac complication rates. However, the ANH group had a lower incidence of dobutamine use (odds ratio, 0.35; P=0.04) and fewer arrhythmias, indicating better hemodynamic stability and less myocardial stress.

Goldberg et al. [23] found that ANH was associated with a reduction in RBC transfusions, with the most significant reduction observed in patients who received 800 mL or more of ANH (RRadj 0.57, P<0.001). In our study, while the overall rate of RBC transfusions was not significantly different between the ANH and control groups (35.22% vs. 28.40%, p = 0.611), the ANH group showed a trend toward reduced transfusion requirements. Both our study and Goldberg et al. found no significant differences in ICU and hospital stay or non-cardiac complication rates between the ANH and control groups. However, the trend toward reduced inotropic support and lower myocardial injury markers in our study suggests that ANH may have a subtle but beneficial effect on postoperative recovery. Goldberg et al. reported superior postoperative morbidity and mortality in the ANH group, which aligns with our findings on myocardial protection and inotropic support. Our results are consistent with and extend the findings of the study by Sebastian et al. [24], which focused on the impact of ANH in pediatric cardiac surgery. Sebastian et al. [24] found that higher ANH volume and longer storage time were associated with a greater need for intraoperative transfusions. However, 27 out of 50 patients (54%) did not require any blood transfusions during their hospitalization.

## Conclusion

Preventing myocardial damage during heart surgery leads to better recovery. In combination with blood cardioplegia and anesthetic preconditioning, acute preoperative hemodilution reduces the detrimental effects of aortic occlusion and improves myocardial recovery in patients undergoing CABG. The beneficial effect of hematocrit levels before cardiopulmonary bypass is unknown. Further studies are needed to confirm these preliminary results, investigate the cardioprotective mechanisms associated with hemodilution, and test the efficacy of this simple procedure in patients with risk factors. Good luck to those with poor ventricular function and those needing cardiac surgery.

## Conflict of Interest

none to be declared.

## AI Disclosure Statement

During the preparation of this manuscript, the authors used ChatGPT, OpenAI company for language editing, grammar improvement, and liboberry.com for reference management. After its use, the authors thoroughly reviewed, verified, and revised all AI-assisted content to ensure accuracy and originality. The authors take full responsibility for the integrity and final content of the published article.

## References

[R1] Kalter RD, Saul CM, Wetstein L, Soriano C, Reiss RF (1979). Cardiopulmonary bypass: associated hemostatic abnormalities. The Journal of Thoracic and Cardiovascular Surgery.

[R2] Doenst T, Bargenda S, Kirov H, Moschovas A, Tkebuchava S, Safarov R, Velichkov I, Diab M (2020). Cardiac surgery 2019 reviewed. The Thoracic and Cardiovascular Surgeon.

[R3] Dutta P, Emani S, Nathan M, Emani S, Ibla JC (2023). Implications of transfusion in adults with congenital heart
disease undergoing cardiac surgery. Pediatric Cardiology.

[R4] Snyder EL, Sekela ME, Welsby IJ, Toyoda Y, Alsammak M, Sodha NR, Beaver TM, Pelletier JP, Gorham JD, McNeil JS, Sniecinski RM (2023). Evaluation of the efficacy and safety of amustaline/glutathione
pathogen-reduced RBCs in complex cardiac surgery: the Red Cell Pathogen
Inactivation (ReCePI) study, protocol for a phase 3, randomized, controlled
trial. Trials.

[R5] Balkan B, Ulubay ZÖ, Güneysu E, Dündar AS, Turan Eİ (2024). Determinants of 30-day mortality in elderly patients admitted to
a cardiovascular surgery intensive care unit. Turkish Journal of Trauma & Emergency Surgery.

[R6] Neto MM, Pereira M, Gatto GC, Libonati LG (2021). Transfusion Strategy and Postoperative Complications in Adults
Undergoing Cardiac and Vascular Surgery: Systematic Review and Meta-analysis. Journal of Advances in Medicine and Medical Research.

[R7] Chazal E, Morin L, Chocron S, Lassalle P, Pili-Floury S, Salomon du, Ferreira D, Samain E, Perrotti A, Besch G (2023). Impact of early postoperative blood glucose variability on serum
endocan level in cardiac surgery patients: a sub study of the ENDOLUNG
observational study. Cardiovascular Diabetology.

[R8] Keeling-Johnson K, Baker D, Want T, Tuazon DM (2023). Immediate Postoperative Management of Cardiac Surgery Patients. Methodist DeBakey Cardiovascular Journal.

[R9] Amatya AG, Sharma A, Shrestha BK, Nepal B, Paneru HR, Shrestha A, Baral R, Gurung A, Panthee N, Gandhi A, Goerlinger K (2023). Patient blood management for cardiovascular surgery: Clinical
practice consensus statement. Nepalese Heart Journal.

[R10] Dhingra N, Verma R, Mazer CD, Verma S (2024). Generalizability of SELECT to a contemporary cohort of cardiac
surgical patients: analysis of the TRICS III Randomized Trial. European Heart Journal.

[R11] Viana P, Relvas JH, Persson M, Cabral TD, Persson JE, de Oliveira, Bonow P, Freire CV, Amaral S (2024). Prothrombin Complex Concentrate versus Fresh Frozen Plasma in
Adult Patients Undergoing Cardiac Surgery: A Systematic Review and
Meta-Analysis. Journal of Chest Surgery.

[R12] Ming Y, Zhang F, Yao Y, Cheng Z, Yu L, Sun D, Sun K, Yu Y, Liu M, Ma L, HuangYang Y (2023). Large volume acute normovolemic hemodilution in patients
undergoing cardiac surgery with intermediate-high risk of transfusion: a
randomized controlled trial. Journal of Clinical Anesthesia.

[R13] Li J, Xia Y, Jin S, Dong H, Zhao P, Jiang H, Hu R (2023). Effects of acute normovolemic hemodilution on allogeneic blood
transfusion & coagulation in orthognathic surgery: A randomized study. Transfusion.

[R14] Jones SB, Whitten CW, Despotis GJ, Monk TG (2003). The influence of crystalloid and colloid replacement solutions in
acute normovolemic hemodilution: a preliminary survey of hemostatic markers. Anesthesia & Analgesia.

[R15] Midorikawa Y, Saito J, Kitayama M, Toyooka K, Hirota K (2021). Intra-operative intravascular effect of the difference in colloid
solutions during acute normovolemic hemodilution. JA Clinical Reports.

[R16] Konrad FM, Mik EG, Bodmer SI, Ates NB, Willems HF, Klingel K, de Geus, Stolker RJ, Johannes T (2013). Acute normovolemic hemodilution in the pig is associated with
renal tissue edema, impaired renal microvascular oxygenation, and functional
loss. Anesthesiology.

[R17] Li S, Liu Y, Zhu Y (2020). Effect of acute normovolemic hemodilution on coronary artery
bypass grafting: a systematic review and meta-analysis of 22 randomized
trials. International Journal of Surgery.

[R18] Barile L, Fominskiy E, Di Tomasso, Castro LE, Landoni G, De Luca, Bignami E, Sala A, Zangrillo A, Monaco F (2017). Acute normovolemic hemodilution reduces allogeneic red blood cell
transfusion in cardiac surgery: a systematic review and meta-analysis of
randomized trials. Anesthesia & Analgesia.

[R19] Casati V, Benussi S, Sandrelli L, Grasso MA, Spagnolo S, D’Angelo A (2004). Intraoperative moderate acute normovolemic hemodilution
associated with a comprehensive blood-sparing protocol in off-pump coronary
surgery. Anesthesia & Analgesia.

[R20] Mahoori A, Heshmati F, Noroozinia H, Mehdizadeh H, Salehi S, Rohani M (2009). Intraoperative minimal acute normovolemic hemodilution in
patients undergoing coronary artery bypass surgery. Middle East Journal of Anaesthesiology.

[R21] Mladinov D, Eudailey KW, Padilla LA, Norman JB, Leahy B, Enslin J, Parker K, Cornelius KF, Davies JE (2021). Effects of acute normovolemic hemodilution on
post‐cardiopulmonary bypass coagulation tests and allogeneic blood
transfusion in thoracic aortic repair surgery: An observational cohort study. Journal of Cardiac Surgery.

[R22] Droz NM, Lin J, Beach J, Vo C, Morrow K, Lyden SP, Hardy DM, Caputo FJ, Rowse J, Kirksey L, Smolock CJ (2021). Decreased transfusion requirements with use of acute normovolemic
hemodilution in open aortic aneurysm repair. Journal of Vascular Surgery.

[R23] Goldberg J, Paugh TA, Dickinson TA, Fuller J, Paone G, Theurer PF, Shann KG, Sundt III, Prager RL, Likosky DS, Registry P (2015). Greater volume of acute normovolemic hemodilution may aid in
reducing blood transfusions after cardiac surgery. The Annals of thoracic surgery.

[R24] Sebastian R, Ratliff T, Winch PD, Tumin D, Gomez D, Tobias J, Galantowicz M, Naguib AN (2017). Revisiting acute normovolemic hemodilution and blood transfusion
during pediatric cardiac surgery: a prospective observational study. Pediatric Anesthesia.

